# Study protocol: Using ecological momentary assessment and wearable sensors to examine mechanisms linking sleep and smoking cessation among adults who are socioeconomically disadvantaged

**DOI:** 10.1371/journal.pone.0334129

**Published:** 2025-10-30

**Authors:** Chaelin K. Ra, Michael Businelle, Karen Gamble, Michael B. Steinberg, Donald Hedeker, Andrea Spaeth, Andrea C. Villanti

**Affiliations:** 1 Rutgers Cancer Institute of New Jersey, Robert Wood Johnson Medical School, Rutgers University, New Jersey, United States of America; 2 Rutgers Institute for Nicotine & Tobacco Studies, Rutgers University, New Jersey, United States of America; 3 TSET Health Promotion Research Center, Stephenson Cancer Center, University of Oklahoma Health Sciences Center, Oklahoma City, Oklahoma, United States of America; 4 Behavioral Neurobiology, Psychiatry, School of Medicine, University of Alabama at Birmingham, Alabama, United States of America; 5 Department of Public Health Sciences, University of Chicago, Illinois, United States of America; 6 Department of Kinesiology and Health, Rutgers University, New Jersey, United States of America; Public Library of Science, UNITED KINGDOM OF GREAT BRITAIN AND NORTHERN IRELAND

## Abstract

**Background:**

Cigarette smoking is highly concentrated among individuals with lower socioeconomic status (SES) who often lack access to smoking cessation services. Thus, smoking cessation in lower SES adults remains a critical public health concern that warrants further study and attention. Smokers attempting to quit are at the highest risk for lapse within the first weeks of their quit attempt, and an initial lapse is highly likely to lead to full relapse. It is essential to identify and understand behavioral factors that may increase or decrease the likelihood of successful smoking cessation among lower SES adults during a quit attempt (pre-and post-quit). Recently, sleep dysregulation, such as insufficient sleep duration, has been considered as a potential intervention target to address smoking behaviors (e.g., number of cigarettes smoked per day) and improve smoking cessation outcomes (e.g., abstinence). Recent studies have found that lower SES is associated with higher rates of poor sleep. Thus, SES should be accounted for when assessing sleep dysregulation during smoking cessation attempts. Although previous studies have examined the relationship between sleep dysregulation and smoking behavior and/or cessation outcomes, they have several methodological limitations, including the use of retrospective survey methods, use of cross-sectional study designs, relying solely on laboratory-based data collection, not assessing integrated sleep health dimensions (usually only sleep duration or quality is assessed), omitting lower SES adults who smoke, and focusing on a single pathway rather than bidirectional associations.

**Methods:**

This study will use a real-time data capture approach among lower SES adults who are attempting to quit smoking. This approach will involve a granular examination of the bidirectional and temporal associations between daily sleep dysregulation and smoking cession processes (pre- and post-quit) using smartphone-based ecological momentary assessment (EMA) and wearable sensors. Specifically, we aim to identify bidirectional and temporal associations between daily smoking abstinence and sleep dysregulation via EMA and wrist-worn sensors during the first four weeks of a smoking cessation attempt.

**Discussion:**

Findings from this study will yield preliminary data that will be used to develop and implement a Just-in-Time-Adaptive Intervention (JITAI) that aims to improve sleep health during smoking cessation.

## Background

This study seeks to elucidate the bidirectional associations between daily sleep dysregulation and smoking cessation among lower socioeconomic status (SES) adults who smoke. Tobacco use disorder is the most common substance use disorder (SUD).[1] Cigarette smoking, the leading preventable cause of morbidity and mortality in the United States, [2,3] is concentrated among individuals with lower SES.[4] Although most adults who smoke express interest in quitting and half make a serious quit attempt each year, [5] adults with lower SES often lack access to evidence-based cessation services and have worse smoking cessation outcomes than higher SES people.[6,7] Thus, smoking cessation in lower SES adults remains a critical public health concern that warrants further study and intervention.

Gaining a better understanding of the associations between sleep dysregulation and smoking behavior and cessation outcomes may enhance smoking cessation interventions for underserved lower SES adults who smoke. Sleep dysregulation increases smoking behavior (e.g., the number of cigarettes per day) and reduces the odds of a successful attempt to quit. Lower SES is associated with poor sleep, [8–15] and for that reason, studies recommend accounting for SES when examining sleep dysregulation.[16] On the other hand, sufficient sleep duration and better sleep quality have been associated with fewer cigarettes smoked per day, [17] less nicotine dependence, [18] lower withdrawal levels, [19] and fewer smoking relapses.[20] Furthermore, there is evidence that cigarette smoking negatively impacts sleep duration and quality.[21] Indeed, adults who smoke tend to experience more sleep disturbance and have less total sleep time than those who do not smoke.[22] Despite documented associations between sleep dysregulation, smoking behavior, and cessation outcomes, previous studies have several limitations, which include: 1) use of retrospective survey methods can result in recall bias, [23–26] 2) a focus on singular dimensions of sleep (usually sleep duration or quality) which limit a full understanding of these complex relationships, [18,19,27–30] 3) use of cross-sectional methods which provides a one-time “snapshot of studied relationships,” [27,31–33] 4) dependence on laboratory-based data collection, which may not reflect smoking in natural environments, [21,34–36] 5) an omission of lower SES individuals, and 6) a focus on a single pathway between sleep and smoking behavior or cessation outcomes rather than bidirectional associations.[19,21,22,37].

To address these gaps in the literature, we propose to use smartphone-based ecological momentary assessment (EMA) surveys and wearable sensors to obtain a more granular understanding of the temporal associations between daily sleep dysregulation and smoking behaviors and cessation outcomes among lower SES adults who are attempting to quit smoking. These methods will utilize daily sleep [38] and smoking measures, and assess moment-to-moment feelings, behaviors, and experiences associated with sleep and cigarette use in free-living settings.[39].

Completion of the proposed research will improve our understanding of bidirectional and temporal associations between daily sleep health and smoking maintenance, withdrawal, abstinence, and relapse processes in lower SES adults attempting to quit smoking. Study findings will inform the development of future evidence-based interventions to enhance sleep health and increase smoking cessation success in people at greatest risk of tobacco-related morbidity and mortality.

## Methods

### Study aim

This study aims to identify bidirectional and temporal associations of daily smoking abstinence and sleep dysregulation via EMA and wrist-worn sensors during the first four weeks of a smoking cessation attempt.

### Study hypotheses

Hypothesis 1. On days when participants are abstinent, they will be more likely to have poorer sleep health (shorter duration, later bedtime, longer latency, lower efficiency) that night due to withdrawal symptoms; however, the effect will be attenuated over time.

Hypothesis 2. On days when participants have slept more poorly (shorter duration, later bedtime, longer latency, lower efficiency), they will be more likely to lapse or relapse to smoking.

### Study design and overview

**Fig 1:**
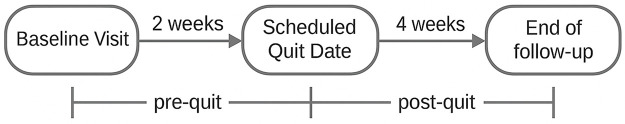
Study overview.

Study participation will last 6 weeks and include 3 virtual visits (10–40 minutes each; baseline, quit date, and end of the study) and 5 daily EMAs for 42 days. After the baseline visit, participants will complete daily EMAs for 2 weeks in the pre-quit period and for 4 weeks in the post-quit period (see Fig 1). Participants will be required to complete EMAs 30 minutes after their preset waking time (morning diary), 3 random times (random sampling) during each day and one hour before bedtime (evening diary), to assess any smoking-related triggers and behaviors and smoking events and to complete biochemical verification of smoking status via iCO Breathlyzer in the evening [40] Participants can also initiate prompts when they smoke. In addition, participants will wear an ActiWatch (Motion Watch 8; CamNTech, Ltd) [41] during the 6-week study period (See Measures section for details). Participants will complete a follow-up assessment 4 weeks after their end of study to assess relevant variables including smoking status.

### Study setting and population

Participants (N = 80) will be adults who smoke cigarettes and attend one of 11 Quitcenters across the state of New Jersey between June 2026 and June 2028. Individuals seeking smoking cessation services at the Quitcenters will be given a verbal description of the study during their first visit, and interested individuals will be screened for study eligibility. Eligibility criteria are: 1) household income <200% of the federal poverty line, [42] 2) a score ≥ 4 on the Rapid Estimate of Adult Literacy in Medicine-Short Form (REALM-SF), [43] 3) willingness to quit smoking 14 days after the baseline visit, 4) ≥ 18 years of age, 5) an expired CO level ≥ 7 ppm, 6) currently smoking ≥ 5 cigarettes per day, 7) no overnight shiftwork, 8) not currently using potentially sedating medications, and 9) no contraindications to using NRT (per self-report). All participants will provide written informed consent prior to participation in the study.

### Procedure

Participants will be provided an Actiwatch, and an iCO Breathalyzer. Participants who do not have a smartphone that meets the minimum criteria to support the Insight^TM^ app [44] will also be provided a phone. Participants will be instructed regarding using the Insight^TM^ smartphone application, Actiwatch, Breathalyzer, and EMA procedures. Participants will complete questionnaires at the baseline visit, including the pre-treatment assessment of sleep, other tobacco use, past/current medical history, provide a CO breath sample to verify smoking status, and measure of neck circumference.

Participants will use the Insight^TM^ app [44] to record subjective sleep and wake times and will wear an Actiwatch to obtain objective sleep data continuously for the entire 6-week study period. At the end of the study, participants will return the Actiwatch and study phone (if they did not use their own smartphone) via mail or curbside drop-off. All participants will download the Insight^TM^ mHealth application [44], which includes a “Call Staff” function/button that automatically calls study staff (e.g., if the participant has problems completing EMAs). The Insight^TM^ app [44] also includes a “Payment” button which, when pressed, indicates the number of EMAs that have been prompted and completed and the current level of compensation based upon the up-to-the-moment percentage of EMAs completed. All participants will wear Actiwatch, complete 3 virtual visits, and complete 5 daily EMAs (i.e., 3 random,1 morning and 1 evening diaries; see Measures section for details). Participants will receive $15 for completing each visit ($45 in total) and will be compensated based upon the percentage of Daily and Random EMAs completed (i.e., $70 for completing 50%−74% of prompted EMAs, $90 for completing 75%−89% of prompted EMAs, and $120 for completing 90% or more prompted EMAs). An additional $50 payment will be awarded to participants for wearing the Actiwatch at least 90% of the time. This payment will be provided after participants return the Actiwatch. There will be additional $10 payment for those who complete the one-month follow-up survey. The use of personal vs. study-provided phones will be examined as a covariate in all analyses. Data collection is expected to be completed by August 2028.

This study was approved by the Rutgers Institutional Review Board (Pro2022002184). All participants will provide written informed consent prior to participation in the study. The consent process includes a full explanation of the study’s aims, procedures, potential risks, and benefits. Participants will be informed of their right to withdraw from the study at any time without any consequence. Confidentiality and anonymity of participants will be maintained throughout the study.

### Measures

**Virtual visits (i.e., baseline, quit date, and end of study):** baseline visit assessments will assess demographic, anthropometric (e.g., self-reported height and weight), sleep-related characteristics (e.g., STOP-BANG [45]), tobacco use characteristics (e.g., use of e-cigarettes), and other mental diagnoses, medical history, tobacco/e-cigarette and other substance use, and multiple constructs related to smoking lapse (e.g., stress, affect, anxiety). Quit date visit assessments will include participant’s readiness to quit, biochemical verification of smoking abstinence, and multiple constructs related to smoking lapse (e.g., stress, affect, anxiety). End of study visit assessments will assess participants’ experience of using the app, biochemical verification of smoking abstinence, and multiple constructs related to smoking lapse (e.g., stress, affect, anxiety).

**EMAs:** Three types of EMA sampling will be used: daily diaries, random sampling, and event sampling. Daily diary assessments will be completed

twice every day (30 minutes after waking and 1 hour before bedtime), and questions will refer to the previous few hours and the current moment (Table 1). The phone audibly and visually cues each EMA. If the participant does not respond after three prompts, the assessment will be recorded as missed. Participants will self-initiate event sampling EMAs. Participants will be instructed to complete “smoking assessments” when they smoke before the quit date and “lapse assessments” if they smoke after the quit date. EMA items will assess smoking (e.g., cigarettes smoked, time since last cigarette), use of NRT, mood (e.g., sadness, happiness), motivation to quit smoking, availability of cigarettes, and social setting. Daily smoking abstinence will be calculated using a standard algorithm from multiple assessments of smoking throughout the EMAs. The Actiwatch [46] will collect data on activity (i.e., arm motion) and ambient light that is related to sleep. Actigraphy data will be downloaded from the actigraphy watch into the Motionware software (version 1.4.25) for analysis, management, and exportation. Total sleep time, sleep start and end times, sleep onset latency, sleep duration, and sleep efficiency will be calculated objectively with a standard algorithm [41]. Mid-sleep time will be calculated as the midpoint between sleep start and end times as an indicator of the circadian phase [41].

**Table 1:** EMA Items of the study.

EMA	MEASURE
Morning Daily Diary	Cigarettes smoked
	Social interactions and support
	NRT use
	Side Effect Items
	Alcohol Consumption
	Sleep Items
End of Day Daily Diary	Cigarettes smoked
	Other substance use
	iCO Breath test
Random/Event EMAs	Recency of smoking
	Urge to smoke
	Stress
	Availability of cigarettes
	Recent alcohol use
	Cessation motivation
	Interaction with smokers
	Social setting/location
	Reasons for lapse
	Cigarette reward value
	Lapse warning signs

### Power

A two-step power analysis was conducted. In step 1, G*Power 3.1.9.2.[47] was used to estimate power for a traditional regression model. Based on our preliminary study, [48] an effect size of *f*^*2*^ = .08 was used for the current study. Using G*Power, it was estimated that a sample of 177 would be needed to observe the effect size (*f*^*2*^ = .08) of sleep duration/quality and smoking abstinence to achieve power of.80 with an alpha level of.05 for six predictors. However, G*Power cannot estimate the sample size for multilevel models (e.g., nested data). Therefore, in step 2, we examined the number of unique observations needed to estimate the accurate sample size for multilevel models given the multilevel structure of the data.[49] West, Ryu, Kwok, and Cham’s (2011) equation used for step 2 is below:


$$N_{effective}=\frac{nL_{1}nL_{2}}{[1+(nL_{1}–1)𝐈𝐂𝐂]}$$

This equation accounts for level 1 (daily diary; nL1) and level 2 (participant; nL2) sample sizes, and the intraclass correlation (ICC) which is the proportion of the variance in the dependent variable that is attributed to variability between people. Based on prior research, a moderate amount of within-person variation is expected for smoking cessation (range ICC = 0.2–0.4). Using this information of the sample size of 42 (7X6) days (Level 1; nL1) and a conservative ICC value of 0.2 from our previous work on sleep and smoking cessation [48] it was estimated that approximately 39 participants (Level 2; nL2) will provide sufficient power. With these estimates the *N*_*effective*_ is 179 independent observations which exceeds the required 177 independent observations from step 1. The sample size of 50 participants or more will provide sufficient power for the proposed study, 50*(42 days) observations; n = 2100 (n = 1680, taking into consideration expected EMA compliance rates of 80%). Trained staff will closely monitor participant compliance rates.

### Analytic approach

**Fig 2:**
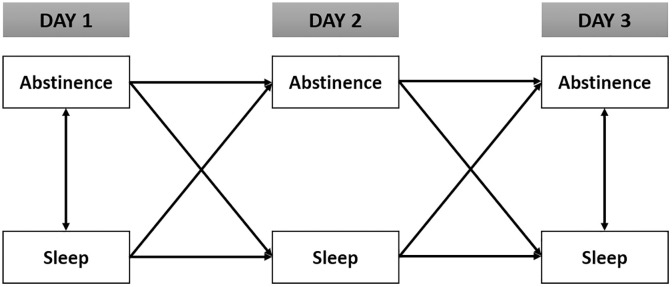
Cross-lagged models for analyzing bidirectional associations..

Descriptive statistics will summarize the distributions of outcomes and demographic characteristics. We will also explore any differences in outcomes by demographic characteristics (e.g., sex, race). If more than 10% of the outcomes are missing, methods for missing data will be considered. To identify bidirectional and temporal associations of daily abstinence and sleep dysregulation via EMA and wrist worn sensors during the first four weeks of a smoking cessation attempt (N = 80), cross-lagged models (see Fig 2) will be tested to examine 1 simultaneously) the association of sleep dysregulation with subsequent changes in daily abstinence, and 2) the association of daily abstinence with subsequent changes sleep dysregulation using Actiwatch. We will use multilevel models with lagged effects in SAS 9.4 software (SAS Institute, Cary, North Carolina) to test the temporal associations between daily sleep dysregulation and smoking abstinence. Two-level models will be conducted with 80 participants who complete the assessments over the 4 weeks of post-quit. The total number of EMAs are estimated to be 1792 ~ 2240 (80% to 100% compliance) clustered within 80 participants. The models will use an AR [1] error covariance structure to account for correlations between consecutive sleep and abstinence observations.[50] When *the outcome variable is smoking abstinence*, either sleep duration, bedtime, sleep latency, or sleep efficiency will be the predictor variable. When *the outcome variable is a nighttime sleep measure* (e.g., duration, sleep maintenance efficiency, sleep onset, sleep offset), daily smoking abstinence will be the predictor variable (alternatively, other cessation outcomes (e.g., urge) will be also tested). Variances for sleep dysregulation and abstinence measures will be decomposed to within-person and between-person components. Within-person variables will be centered at the person mean, so that positive values indicate scores higher than the person’s cross-day average. Between-person variables will be centered at the sample mean so that positive values indicate higher scores than others in the sample. A stepwise procedure will be used for variable selection, and variables from the procedure (e.g., sex, ethnicity, age, other tobacco/e-cigarette use, mental diagnosis, NRT use, BMI) will be included as covariates in all models. In addition, sensitivity analysis will be conducted to evaluate the robustness of the results from the analyses. We attend to sex as a biological variable by: 1) recruiting both sexes, 2) stratifying randomization by sex, and 3) exploring any differences in outcomes by sex.

## Discussion

Sleep dysregulation is an understudied phenomenon and underappreciated intervention target for promoting smoking cessation and preventing relapse among smokers who want to quit.[20] Although several studies have demonstrated associations between sleep dysregulation and smoking behavior and cessation outcomes, limitations include using retrospective [1] or cross-sectional [27,31–33] survey methods focusing on singular sleep dimensions (usual sleep duration or quality), [1] testing a simple comparison (e.g., smokers vs. non-smokers, pre/post-tests) or using laboratory-based data collection, [21,34–36] and exclude lower SES adults who smoke. Thus, these studies provide only an “artificial lab-based” snapshot of behavior and focus on a single pathway. Our proposed study addresses these gaps by conducting a prospective, observational study to examine the bi-directional and temporal relationships between multiple dimensions of daily sleep dysregulation with smoking behavior and cessation outcomes during the quit attempt (both pre- and post-quit). Using repeated daily sleep measurements (i.e., objective sensor data via Actiwatch and subjective measures via daily diaries) and smoking-related variables, our study will provide a more granular understanding of the complex relationships between these variables in the real world. Aligned with NIDA Notice of Special Interest (NOSI), “Sleep and Substance Use Disorders,” this study will determine the mechanisms of the directionality of sleep dysregulation and smoking maintenance, withdrawal, abstinence, and relapse processes and will illuminate potential treatment targets for prevention and intervention, as they occur in real-time within natural settings for lower SES smokers.

A potential limitation of this study is that asking participants to complete EMAs may serve as an intervention (e.g., self-monitoring alone may improve health outcomes [51]. However, multiple EMA studies that have examined substance use disorders have found that EMA methods result in little to no reactivity.[52–54] A recent study of reactivity in smokers seeking cessation treatment found that frequent EMAs were related to lower craving, anxiety, anger, hunger, and positive affect but were unrelated to abstinence.[55] Importantly, all participants will receive equal numbers of EMAs in this observational study, and those variables will be centered at the person mean. Another limitation may be the burden of the 6-week EMAs and wearing of an Actiwatch. To mitigate this burden, we will use a watch with a long-lasting battery that does not require recharging during the 6-week study period. Additionally, sleep may be affected by the current season and data collection will span all four seasons (i.e., spring, summer, fall, and winter).[56,57] This study will include time of year as a covariate to avoid the potentially confounding effect of season. We indicated alternative strategies for secondary analysis using alternative outcomes (e.g., urge, cravings) for the study.

The proposed project will be among the first to examine associations between sleep dysregulation and smoking behavior and cessation outcomes using intensive longitudinal data via EMA and wearable sensors during a scheduled smoking cessation attempt. Improving sleep health in people attempting to quit smoking has emerged as a potential non-pharmacological candidate to improve cessation outcomes.[20] This project will provide preliminary data for a future R01 study focused on providing just-in-time adaptive sleep interventions (e.g., tailored messages) among lower SES adults who are attempting to quit smoking. For instance, Cognitive Behavior Therapy-based (CBT) just-in-time adaptive interventions (JITAI) that integrate sleep hygiene components [58] (e.g., maintaining a regular sleep routine, mindfulness training prior to the quit attempt, and/or CBT for sleep deprivation during cessation may be used to improve sleep health, which in turn may improve cessation outcomes. In addition, future studies may integrate other health behaviors that may mediate and/or moderate sleep dysregulation, smoking behavior, and cessation outcomes (e.g., physical activity or other substance use).

## Supporting information

S1 Table Planned EMA questionnaire. (DOCX)

## Abbreviations

SES: Socioeconomic Status
EMA: Ecological Momentary Assessment
REALM-SF: Rapid Estimate of Adult Literacy in Medicine-Short Form
CO: Carbon Monoxide
NRT: Nicotine Replacement Therapy
GLMM: Generalized Linear Mixed Models
ICC: Intraclass Correlation Coefficient
SAS: Statistical Analysis System
AR(1): Autoregressive Errors
JITAI: Just-in-Time-Adaptive Interventions
CBT: Cognitive Behavior Therapy.

## References

[1] Weinberger AH, Platt J, Esan H, Galea S, Erlich D, Goodwin RD. Cigarette Smoking Is Associated With Increased Risk of Substance Use Disorder Relapse: A Nationally Representative, Prospective Longitudinal Investigation. J Clin Psychiatry. 2017;78(2):e152–60. doi: 10.4088/JCP.15m10062 28234432 PMC5800400

[2] Prochaska JJ, Das S, Young-Wolff KC. Smoking, Mental Illness, and Public Health. Annu Rev Public Health. 2017;38:165–85. doi: 10.1146/annurev-publhealth-031816-044618 27992725 PMC5788573

[3] Gfroerer J, Dube SR, King BA, Garrett BE, Babb S, McAfee T. Vital signs: current cigarette smoking among adults aged≥ 18 years with mental illness—United States, 2009–2011. MMWR Morbidity and mortality weekly report. 2013;62(5):81. 23388551 PMC4604817

[4] Jamal A, King BA, Neff LJ, Whitmill J, Babb SD, Graffunder CM. Current Cigarette Smoking Among Adults - United States, 2005-2015. MMWR Morb Mortal Wkly Rep. 2016;65(44):1205–11. doi: 10.15585/mmwr.mm6544a2 27832052

[5] Wang TW, Asman K, Gentzke AS, Cullen KA, Holder-Hayes E, Reyes-Guzman C, et al. Tobacco Product Use Among Adults - United States, 2017. MMWR Morb Mortal Wkly Rep. 2018;67(44):1225–32. doi: 10.15585/mmwr.mm6744a2 30408019 PMC6223953

[6] Schroeder SA, Morris CD. Confronting a neglected epidemic: tobacco cessation for persons with mental illnesses and substance abuse problems. Annu Rev Public Health. 2010;31:297-314 1p following 314. doi: 10.1146/annurev.publhealth.012809.103701 20001818

[7] Williams JM, Zimmermann MH, Steinberg ML, Gandhi KK, Delnevo C, Steinberg MB, et al. A comprehensive model for mental health tobacco recovery in new jersey. Adm Policy Ment Health. 2011;38(5):368–83. doi: 10.1007/s10488-010-0324-x 21076862 PMC3638154

[8] Grandner MA, Patel NP, Gehrman PR, Xie D, Sha D, Weaver T, et al. Who gets the best sleep? Ethnic and socioeconomic factors related to sleep complaints. Sleep Med. 2010;11(5):470–8. doi: 10.1016/j.sleep.2009.10.006 20388566 PMC2861987

[9] Xiao Q, Hale L. Neighborhood socioeconomic status, sleep duration, and napping in middle-to-old aged US men and women. Sleep. 2018;41(7):zsy076. doi: 10.1093/sleep/zsy076 29697844 PMC6047422

[10] Hale L, Do DP. Racial differences in self-reports of sleep duration in a population-based study. Sleep. 2007;30(9):1096–103. doi: 10.1093/sleep/30.9.1096 17910381 PMC1978399

[11] Hale L. Who has time to sleep?. J Public Health (Oxf). 2005;27(2):205–11. doi: 10.1093/pubmed/fdi004 15749721

[12] Lauderdale DS, Knutson KL, Yan LL, Rathouz PJ, Hulley SB, Sidney S, et al. Objectively measured sleep characteristics among early-middle-aged adults: the CARDIA study. Am J Epidemiol. 2006;164(1):5–16. doi: 10.1093/aje/kwj199 16740591

[13] Patel SR. Social and demographic factors related to sleep duration. Sleep. 2007;30(9):1077–8. doi: 10.1093/sleep/30.9.1077 17910376 PMC1978396

[14] Patel SR, Malhotra A, Gottlieb DJ, White DP, Hu FB. Correlates of long sleep duration. Sleep. 2006;29(7):881–9. doi: 10.1093/sleep/29.7.881 16895254 PMC3500381

[15] Adams J. Socioeconomic position and sleep quantity in UK adults. J Epidemiol Community Health. 2006;60(3):267–9. doi: 10.1136/jech.2005.039552 16476759 PMC2465560

[16] Jehan S, Myers AK, Zizi F, Pandi-Perumal SR, Jean-Louis G, Singh N, et al. Sleep health disparity: the putative role of race, ethnicity and socioeconomic status. Sleep Med Disord. 2018;2(5):127–33. doi: 10.15406/smdij.2018.02.00057 31179440 PMC6553614

[17] Warren CM, Riggs NR, Pentz MA. Longitudinal relationships of sleep and inhibitory control deficits to early adolescent cigarette and alcohol use. J Adolesc. 2017;57:31–41. doi: 10.1016/j.adolescence.2017.03.003 28334632 PMC5436806

[18] Cohen A, Ben Abu N, Haimov I. The Interplay Between Tobacco Dependence and Sleep Quality Among Young Adults. Behav Sleep Med. 2020;18(2):163–76. doi: 10.1080/15402002.2018.1546707 30463440

[19] Purani H, Friedrichsen S, Allen AM. Sleep quality in cigarette smokers: Associations with smoking-related outcomes and exercise. Addict Behav. 2019;90:71–6. doi: 10.1016/j.addbeh.2018.10.023 30368021 PMC6324958

[20] Patterson F, Grandner MA, Malone SK, Rizzo A, Davey A, Edwards DG. Sleep as a Target for Optimized Response to Smoking Cessation Treatment. Nicotine Tob Res. 2019;21(2):139–48. doi: 10.1093/ntr/ntx236 29069464 PMC6329404

[21] Bender AM, Van Dongen HPA, Roll JM, Layton ME. Sleep disturbance and daytime sleepiness in cigarette smokers attempting to quit without treatment. Sleep Biol Rhythms. 2019;18(1):9–16. doi: 10.1007/s41105-019-00235-y

[22] Costa M, Esteves M. Cigarette Smoking and Sleep Disturbance. Addictive Disorders & Their Treatment. 2018;17(1):40–8. doi: 10.1097/adt.0000000000000123

[23] Arrazola RA, Ahluwalia IB, Pun E, de Quevedo IG, Babb S, Armour BS. Current tobacco smoking and desire to quit smoking among students aged 13–15 years—global youth tobacco survey, 61 countries, 2012–2015. MMWR Morbidity and mortality weekly report. 2017;66(20):533. 28542119 10.15585/mmwr.mm6620a3 PMC5657874

[24] Grant BF, Hasin DS, Chou SP, Stinson FS, Dawson DA. Nicotine dependence and psychiatric disorders in the United States: results from the national epidemiologic survey on alcohol and related conditions. Arch Gen Psychiatry. 2004;61(11):1107–15. doi: 10.1001/archpsyc.61.11.1107 15520358

[25] Lawrence D, Mitrou F, Zubrick SR. Non-specific psychological distress, smoking status and smoking cessation: United States National Health Interview Survey 2005. BMC Public Health. 2011;11:256. doi: 10.1186/1471-2458-11-256 21513510 PMC3107796

[26] McClave AK, McKnight-Eily LR, Davis SP, Dube SR. Smoking characteristics of adults with selected lifetime mental illnesses: results from the 2007 National Health Interview Survey. Am J Public Health. 2010;100(12):2464–72. doi: 10.2105/AJPH.2009.188136 20966369 PMC2978196

[27] Cohrs S, Rodenbeck A, Riemann D, Szagun B, Jaehne A, Brinkmeyer J, et al. Impaired sleep quality and sleep duration in smokers-results from the German Multicenter Study on Nicotine Dependence. Addict Biol. 2014;19(3):486–96. doi: 10.1111/j.1369-1600.2012.00487.x 22913370

[28] Rapp K, Buechele G, Weiland SK. Sleep duration and smoking cessation in student nurses. Addict Behav. 2007;32(7):1505–10. doi: 10.1016/j.addbeh.2006.11.005 17182193

[29] Dugas EN, Sylvestre MP, O’Loughlin EK, Brunet J, Kakinami L, Constantin E, et al. Nicotine dependence and sleep quality in young adults. Addict Behav. 2017;65:154–60. doi: 10.1016/j.addbeh.2016.10.020 27816041

[30] Peltier MR, Lee J, Ma P, Businelle MS, Kendzor DE. The influence of sleep quality on smoking cessation in socioeconomically disadvantaged adults. Addict Behav. 2017;66:7–12. doi: 10.1016/j.addbeh.2016.11.004 27855299

[31] Branstetter SA, Horton WJ, Mercincavage M, Buxton OM. Severity of Nicotine Addiction and Disruptions in Sleep Mediated by Early Awakenings. Nicotine Tob Res. 2016;18(12):2252–9. doi: 10.1093/ntr/ntw179 27613886

[32] Phillips BA, Danner FJ. Cigarette smoking and sleep disturbance. Arch Intern Med. 1995;155(7):734–7. doi: 10.1001/archinte.1995.00430070088011 7695462

[33] McNamara JPH, Wang J, Holiday DB, Warren JY, Paradoa M, Balkhi AM, et al. Sleep disturbances associated with cigarette smoking. Psychol Health Med. 2014;19(4):410–9. doi: 10.1080/13548506.2013.832782 24040938

[34] Jaehne A, Unbehaun T, Feige B, Cohrs S, Rodenbeck A, Schütz A-L, et al. Sleep changes in smokers before, during and 3 months after nicotine withdrawal. Addict Biol. 2015;20(4):747–55. doi: 10.1111/adb.12151 24797355

[35] Moreno-Coutiño A, Calderón-Ezquerro C, Drucker-Colín R. Long-term changes in sleep and depressive symptoms of smokers in abstinence. Nicotine Tob Res. 2007;9(3):389–96. doi: 10.1080/14622200701188901 17365770

[36] Wetter DW, Fiore MC, Baker TB, Young TB. Tobacco withdrawal and nicotine replacement influence objective measures of sleep. J Consult Clin Psychol. 1995;63(4):658–67. doi: 10.1037//0022-006x.63.4.658 7673544

[37] Jaehne A, Unbehaun T, Feige B, Lutz UC, Batra A, Riemann D. How smoking affects sleep: a polysomnographical analysis. Sleep Med. 2012;13(10):1286–92. doi: 10.1016/j.sleep.2012.06.026 23026505

[38] Willetts M, Hollowell S, Aslett L, Holmes C, Doherty A. Statistical machine learning of sleep and physical activity phenotypes from sensor data in 96,220 UK Biobank participants. Sci Rep. 2018;8(1):7961. doi: 10.1038/s41598-018-26174-1 29784928 PMC5962537

[39] Shiffman S, Stone AA, Hufford MR. Ecological momentary assessment. Annu Rev Clin Psychol. 2008;4:1–32. doi: 10.1146/annurev.clinpsy.3.022806.091415 18509902

[40] Adkins J, Dutta P. Monoxalyze: verifying smoking cessation with a keychain-sized carbon monoxide breathalyzer. In: Proceedings of the 14th ACM Conference on Embedded Network Sensor Systems CD-ROM, 2016.

[41] MotionWatch 8.: CamNTech Ltd.; [cited 2025 February 26]. Available from: https://www.camntech.com/motionwatch-8/

[42] UDo HS, Services H. Annual update of the HHS poverty guidelines. Federal Register. 2009;74(14):4199–201.

[43] Arozullah AM, Yarnold PR, Bennett CL, Soltysik RC, Wolf MS, Ferreira RM, et al. Development and validation of a short-form, rapid estimate of adult literacy in medicine. Med Care. 2007;45(11):1026–33. doi: 10.1097/MLR.0b013e3180616c1b 18049342

[44] TSET Health Promotion Research Center: Mobile Health Technology 2024; [cited 2024. Available from: https://healthpromotionresearch.org/MHealth

[45] Chung F, Subramanyam R, Liao P, Sasaki E, Shapiro C, Sun Y. High STOP-Bang score indicates a high probability of obstructive sleep apnoea. Br J Anaesth. 2012;108(5):768–75. doi: 10.1093/bja/aes022 22401881 PMC3325050

[46] Roomkham S, Hittle M, Cheung J, Lovell D, Mignot E, Perrin D. Sleep monitoring with the Apple Watch: comparison to a clinically validated actigraph. F1000Res. 2019;8:754. doi: 10.12688/f1000research.19020.1

[47] Faul F, Erdfelder E, Buchner A, Lang A-G. Statistical power analyses using G*Power 3.1: tests for correlation and regression analyses. Behav Res Methods. 2009;41(4):1149–60. doi: 10.3758/BRM.41.4.1149 19897823

[48] Ra CK, Hebert E, Frank-Pearce S, Moisiuc R, Alexander A, Kendzor DE, et al., editors. Daily reports of sleep and smoking abstinence: an ecological momentary assessment study. annals of behavioral medicine; 2020: oxford univ press inc journals dept, 2001 EVANS RD, CARY, NC 27513 USA.

[49] West SG, Ryu E, Kwok O-M, Cham H. Multilevel modeling: current and future applications in personality research. J Pers. 2011;79(1):2–50. doi: 10.1111/j.1467-6494.2010.00681.x 21223263

[50] Lee S, Crain TL, McHale SM, Almeida DM, Buxton OM. Daily antecedents and consequences of nightly sleep. J Sleep Res. 2017;26(4):498–509. doi: 10.1111/jsr.12488 28008673 PMC5481508

[51] Compernolle S, DeSmet A, Poppe L, Crombez G, De Bourdeaudhuij I, Cardon G, et al. Effectiveness of interventions using self-monitoring to reduce sedentary behavior in adults: a systematic review and meta-analysis. Int J Behav Nutr Phys Act. 2019;16(1):63. doi: 10.1186/s12966-019-0824-3 31409357 PMC6693254

[52] Hufford MR, Shields AL, Shiffman S, Paty J, Balabanis M. Reactivity to ecological momentary assessment: an example using undergraduate problem drinkers. Psychol Addict Behav. 2002;16(3):205–11. doi: 10.1037//0893-164x.16.3.205 12236455

[53] Litt MD, Cooney NL, Morse P. Ecological momentary assessment (EMA) with treated alcoholics: methodological problems and potential solutions. Health Psychol. 1998;17(1):48–52. doi: 10.1037//0278-6133.17.1.48 9459069

[54] Simpson TL, Kivlahan DR, Bush KR, McFall ME. Telephone self-monitoring among alcohol use disorder patients in early recovery: a randomized study of feasibility and measurement reactivity. Drug Alcohol Depend. 2005;79(2):241–50. doi: 10.1016/j.drugalcdep.2005.02.001 16002033

[55] McCarthy DE, Minami H, Yeh VM, Bold KW. An experimental investigation of reactivity to ecological momentary assessment frequency among adults trying to quit smoking. Addiction. 2015;110(10):1549–60. doi: 10.1111/add.12996 26011583 PMC4565778

[56] Friborg O, Bjorvatn B, Amponsah B, Pallesen S. Associations between seasonal variations in day length (photoperiod), sleep timing, sleep quality and mood: a comparison between Ghana (5°) and Norway (69°). J Sleep Res. 2012;21(2):176–84. doi: 10.1111/j.1365-2869.2011.00982.x 22074234

[57] Wescott DL, Soehner AM, Roecklein KA. Sleep in seasonal affective disorder. Curr Opin Psychol. 2020;34:7–11. doi: 10.1016/j.copsyc.2019.08.023 31536962 PMC8733859

[58] Marciniak MA, Shanahan L, Rohde J, Schulz A, Wackerhagen C, Kobylińska D, et al. Standalone smartphone cognitive behavioral therapy-based ecological momentary interventions to increase mental health: narrative review. JMIR Mhealth Uhealth. 2020;8(11):e19836. doi: 10.2196/19836 33180027 PMC7691088

