# Supplementary material for: Study protocol: Using ecological momentary assessment and wearable sensors to examine mechanisms linking sleep and smoking cessation among adults who are socioeconomically disadvantaged
Source: PLoS One. 2025 Oct 30;20(10):e0334129. doi: 10.1371/journal.pone.0334129 (PMC12574915; doi:10.1371/journal.pone.0334129)
Supplement: S1 Table — (DOCX) [file pone.0334129.s001.docx]

**Supporting Information**

**S1 Table. Planned EMA Questionnaire.**

| **EMA Type** | **Question** | **Response Options** |
| --- | --- | --- |
| Pre-Quit Morning EMAs | At what time did you go to bed last night? | __:___ (AM/PM) |
| Pre-Quit Morning EMAs | After settling down, how long (hours/minutes) did it take you to fall asleep? | __hours ___minutes |
| Pre-Quit Morning EMAs | After falling asleep, about how many times did you wake up in the night? | wake up in the night? 1-10 More than 10 (Specify:_) |
| Pre-Quit Morning EMAs | After falling asleep, for how long (hours/minutes) were you awake during the night in total? | __hours ___minutes |
| Pre-Quit Morning EMAs | At what time did you finally wake up? | __:___ (AM/PM) |
| Pre-Quit Morning EMAs | At what time did you get up? | __:___ (AM/PM) |
| Pre-Quit Morning EMAs | How long (hours/minutes) did you spend in bed last night from first getting in to finally getting up? | __hours ___minutes |
| Pre-Quit Morning EMAs | How would you rate the quality of your sleep last night? | Very Poor  Poor Fair  Good Very Good |
| Pre-Quit Morning EMAs | Did you smoke any cigarettes after the evening survey? | Yes  No |
| Pre-Quit Morning EMAs | If yes, how many cigarettes did you smoke before **waking up** this morning? | 1-20 |
| Pre-Quit Morning EMAs | If more than 20, please specify how many cigarettes. Please do not include more than 2 digits in your answer (i.e., do not write "25 cigarettes", instead just write: "25") | __ |
| Pre-Quit Morning EMAs | If yes, how many cigarettes did you smoke after getting up this morning? | 1-20 |
| Pre-Quit Morning EMAs | If more than 20, please specify how many cigarettes. Please do not include more than 2 digits in your answer (i.e., do not write "25 cigarettes", instead just write: "25") |  |
| Pre-Quit Morning EMAs | I used the following smoking cessation aids after the evening survey yesterday: | Chantix/Varenicline Zyban/Wellbutrin (bupropion) Nicotine Patch Nicotine Gum or Lozenge Nasal Spray Other (Specify:__________) None of the above |
| Pre-Quit Morning EMAs | Yesterday, I took prescription medication for the following disorders: (check all that apply) | Depression  Schizophrenia  Bipolar Disorder  Anxiety Disorder Post Traumatic Stress Disorder (PTSD)  Other (Specify:__________) None of the above |
| Pre-Quit Morning EMAs | Are you ready to quit smoking today? | Yes  No |
| Pre-Quit Morning EMAs | Right now, how MOTIVATED are you to be a non-smoker? | Not at all A little  Moderately  Quite a bit  Extremely |
| Pre-Quit Random EMAs | Did you smoke a cigarette since the last prompt? | Yes  No |
| Pre-Quit Random EMAs | If yes, how long ago did you last smoke a cigarette? | I have not smoked today (not even a puff)  0-15 minutes ago  16-30 minutes ago 31 minutes to 45 minutes ago 46 minutes to 1 hour ago  …continues to 4 hours |
| Pre-Quit Random EMAs | If more than 4 hours, please specify how many hours. | _ hours |
| Pre-Quit Random EMAs | Mark the response that most applies to you RIGHT NOW:  I have an urge to smoke.  I feel stressed.  Cigarettes are available to me.  I am motivated to AVOID smoking.  I am able to cope with my stress right now.  I feel irritable.  I feel restless.  I feel alert. | Strongly disagree Disagree  Neutral  Agree Strongly agree |
| Pre-Quit Random EMAs | Are you interacting with anyone who is smoking? Yes No | Yes  No |
| Pre-Quit Random EMAs | I drank alcohol within the last hour. | Yes  No |
| Pre-Quit Random EMAs | Rate your current level of depression (feeling sad). | Low--------------------------------- High (Place a mark on the scale above) |
| Pre-Quit Random EMAs | Rate your current level of anxiety (feeling nervous). | Low--------------------------------- High (Place a mark on the scale above) |
| Pre-Quit Random EMAs | Rate your current level of contentment (feeling happy). | Low--------------------------------- High (Place a mark on the scale above) |
| Pre-Quit Random EMAs | Rate your current level of pain. | Low--------------------------------- High (Place a mark on the scale above) |
| Pre-Quit Random EMAs | Rate your current level of tiredness (lack of energy). | Low--------------------------------- High (Place a mark on the scale above) |
| Pre-Quit Random EMAs | How likely is it that you will smoke between now and the end of the day? | There is a 0 percent chance  There is a 25 percent chance  There is a 50 percent chance  There is a 75 percent chance  There is a 100 percent chance |
| Pre-Quit Random EMAs | Where are you right now? | Home Work Bar/Restaurant Friend's Home Family Member's Home Car/Truck Store Other |
| Pre-Quit Random EMAs | If “Other”, where are you right now? | _______________ |
| Pre-Quit Evening EMAs | Right now, how MOTIVATED are you to be a non-smoker? | Not at all A little  Moderately  Quite a bit  Extremely |
| Pre-Quit Evening EMAs | How likely is it that you WILL quit smoking within the NEXT MONTH? |  |
| Pre-Quit Evening EMAs | How many cigarettes have you smoked today? Please | RESPONSE OPTIONS (1~20) |
| Pre-Quit Evening EMAs | If more than 20, please specify how many cigarettes. | __ cigarettes |
| Pre-Quit Evening EMAs | Please do not include more than 2 digits in your answer | (i.e., do not write “25 cigarettes”, instead just write: “25”) |
| Pre-Quit Evening EMAs | How many standard drinks of alcohol did you have I did not have any drinks today today?  A standard drink is a 12 ounce beer (a 24 ounce beer =2 standard drinks), a 5 ounce glass of wine, or a shot of liquor | I did not have any drinks today  1-10 11 or more: Specify______ |
| Pre-Quit Evening EMAs | I am confident that I can refrain from smoking tomorrow even it is difficult. | Low--------------------------------- High (Place a mark on the scale above) |
| Pre-Quit Evening EMAs | In the past 24 hours, which of the following have you Smoked marijuana used? (select all that apply) | Vaped marijuana Edible marijuana Used marijuana some other way Vaped nicotine Other types of nicotine besides cigarettes, nicotine patches and gum/lozenges None of the above  Other |
| Pre-Quit Evening EMAs | Please describe the other. | __________________ |
| Pre-Quit Evening EMAs | Bedfont iCO assessment will be prompted at the end of the Pre-quit Daily Diary every day.  Step 1. Connect the iCO device to this smartphone.  Step 2. Take a breath and hold it until the timer tells you to exhale. Hold the phone in a manner consistent with taking a selfie while completing the assessment Step 3. Exhale until the timer tells you to stop. Step 4. CO is recorded in the smartphone database. | (Instruction Only) |
| Pre-Quit Evening EMAs | How many naps did you take today? 0 | 1 2 3 4 5 6 or more (Specify:____) |
| Pre-Quit Participant-Initiated EMAs | Approximately how long did you nap in total today (in hours and minutes)? | (i.e., do not write "1 hour 25 minutes", instead just write: "1:25") _____________________________ Bedfont iCO assessment will be prompted at the end of the Pre-quit Daily Diary every day. Step 1. Connect the iCO device to this smartphone Step 2. Take a breath and hold it until the timer tells you to exhale Step 3. Exhale until the timer tells you to stop Step 4. CO is recorded in the smartphone database Participant ID: |
| Pre-Quit Participant-Initiated EMAs | How many cigarettes did you just smoke? | I have not smoked in the past 30 minutes.  1…20  More than 20 (Specify:___) |
| Pre-Quit Participant-Initiated EMAs | Today, how long ago did you last smoke a cigarette? | I have not smoked today (not even a puff) 0-15 minutes ago 16-30 minutes ago 31 minutes to 45 minutes ago 46 minutes to 1 hour ago …continues to 4 hours |
| Pre-Quit Participant-Initiated EMAs | If more than 4 hours, please specify how many hours. | __ hours |
| Pre-Quit Participant-Initiated EMAs | Did you change location in order to smoke? | Yes  No |
| Pre-Quit Participant-Initiated EMAs | Please select the following smoking location that most accurately applies: | (Outdoor area (park, storefront, parking lot, etc)) … 6 (Other) |
| Pre-Quit Participant-Initiated EMAs | Please describe “Other” | ______________ |
| Pre-Quit Participant-Initiated EMAs | Mark the response that most applies to the cigarettes you just SMOKED.  Smoking was pleasurable.  While smoking, I enjoyed the sensations in my throat and chest.  Smoking improved my mood. | Strongly disagree  Disagree  Neutral  Agree  Strongly agree |
| Additional Post-Quit Morning EMAs | Was your most recent cigarette flavored to taste like mint/menthol? | Yes  No |
| Additional Post-Quit Morning EMAs | Yesterday, I was tempted to smoke... (check all that apply) | Out of habit or routine  Because I had a strong craving to smoke Because I saw something that reminded me of smoking Because I wanted to feel more relaxed Because I wanted to socialize with other people who were smoking Actually, I was not tempted to smoke yesterday |
| Additional Post-Quit Morning EMAs | Did anyone offer you a cigarette yesterday? | Yes  No |
| Additional Post-Quit Morning EMAs | Are you ready to quit smoking today? Yes No | Yes  No |
| Additional Post-Quit Evening EMAs | How LIKELY is it that you WILL quit smoking within the NEXT MONTH? | Likert 1 (Not at all) - 5 (Extremely) Agree Neutral Disagree Strongly disagree |
| Additional Post-Quit Evening EMAs | I am confident that I can refrain from smoking tomorrow even if it is difficult. | Strongly agree  Agree Neutral  Disagree Strongly disagree |
| Additional Post-Quit Participant Initiated EMAs | Looking back, how many hours before you smoked did you have warning signs that you might lapse? | 0 – I had NO signs that I would lapse  1 hour ago  …More than 24 hours ago (Specify:__ hours ago) |
| Additional Post-Quit Participant Initiated EMAs | Improving which skills would help you to stay quit in the future?  (check all that apply) | Coping with urges or cravings to smoke  Coping with stress  Coping with other people smoking Coping with arguments Coping with frustration or anger Coping with restlessness Coping with depression Learning more about the harms of smoking Other skills that are not listed Learning how to get better sleep |
| Additional Post-Quit Participant Initiated EMAs | Please type which "other skills" would help you to stay quit in the future. | _____________________________ |
